# Yolk–Shell‐Structured Aluminum Phenylphosphonate Microspheres with Anionic Core and Cationic Shell

**DOI:** 10.1002/advs.201500363

**Published:** 2016-02-25

**Authors:** Liqiu Zhang, Kun Qian, Xupeng Wang, Fan Zhang, Xin Shi, Yijiao Jiang, Shaomin Liu, Mietek Jaroniec, Jian Liu

**Affiliations:** ^1^ Institute of Chemistry for Functionalized MaterialsSchool of Chemistry and Chemical EngineeringLiaoning Normal University850 Huanghe RoadDalian116029P.R. China; ^2^ School of Biomedical EngineeringShanghai Jiao Tong University Med‐X Research Institute1954 Huashan RoadXuhui DistrictShanghai200030P.R. China; ^3^ Department of Chemical EngineeringCurtin UniversityPerthWestern Australia6845Australia; ^4^ Department of Chemistry and BiochemistryKent State UniversityKentOH44242USA

**Keywords:** adsorption, aluminum phenylphosphonate, mesoporous materials, microspheres, yolk‐shell structure

## Abstract

Spherical materials with yolk‐shell structure have great potential for a wide range of applications. The main advantage of the yolk‐shell geometry is the possibility of introducing different chemical or physical properties within a single particle. Here, a one‐step hydrothermal synthesis route for fabricating amphoteric yolk‐shell structured aluminum phenylphosphonate microspheres using urea as the precipitant is proposed. The resulting microspheres display 3D sphere‐in‐sphere architecture with anionic core and cationic shell. The controllable synthesis of aluminum phosphates with various morphologies is also demonstrated. The anionic core and cationic shell of the aluminum phenylphosphonate microspheres provide docking sites for selective adsorption of both cationic methylene blue and anionic binuclear cobalt phthalocyanine ammonium sulphonate. These new adsorbents can be used for simultaneous capture of both cations and anions from a solution, which make them very attractive for various applications such as environmental remediation of contaminated water.

## 1 Introduction

Porous microspheres are of great interest for a variety of applications, such as delivery vehicles, catalysts, adsorbents, and electrodes for energy storage.^1^ Among them, yolk‐shell microspheres represent a new generation of smart functional nanomaterials. The yolk‐shell nanoparticles (YSNs) have distinctive core@void@shell structures and tunable functionalities in both the cores and hollow shells, which make them attractive for a spectrum of applications and inspirational for extensive research on their design and synthesis.^2^ In the past ten years, various YSNs with mesoporous shells and sizes ranging from nanometers to micrometers have been fabricated by soft‐templating, selective‐etching, Ostwald ripening, and ship‐in‐bottle methods.^3^ However, most of these methods involved multistep approaches, and the resultant composition of these YSNs was limited to silica, polymer, carbon, and few metal oxides (e.g., TiO_2_, SnO_2_).^4^, ^5^ Furthermore, it is very challenging to precisely synthesize YSNs with incompatible cores and shells. By adapting an organosilane‐assisted etching method, Yang et al. demonstrated the fabrication of YSNs with a basic core and an acidic shell.^5^ To enrich the composition of yolk‐shell particles, the synthesis of Janus‐type YSNs with different chemical or physical properties within a single particle would be desirable, in particular, by using a facile one‐step method.

As one of the representative organic–inorganic hybrid materials, mesoporous metal phosphonates can be easily tailored by selecting appropriate metal and phosphonic acid to combine different functionalities into one, featuring unique physicochemical properties suitable for various applications ranging from catalysis, separation, adsorption to drug delivery.^6^ Until now, mesoporous metal phosphonate hybrids with various compositions, morphologies, and porosities have been reported;^7^ in particular, mesoporous metal phosphonates with spherical morphology^8^ were synthesized through different methods such as the aerosol method. It is very challenging to precisely control the spherical morphology of mesoporous metal phosphonates due to the easy hydrolysis of metal precursors as well as the rapid precipitation of metal phosphonates.

Herein, we report for the first time the design and synthesis of amphoteric yolk‐shell structured microspheres of aluminum phenylphosphonate using a template‐free hydrothermal synthesis method with urea as the precipitant. Making use of the gradual hydrolysis of urea in the synthesis system, the morphology of the resultant aluminum phenylphosphonates could be modulated by varying the reaction time.

## 2 Results and Discussions

### 2.1 Synthesis and Characterization of Aluminum Phenylphosphonate Microspheres

**Figure 1:**
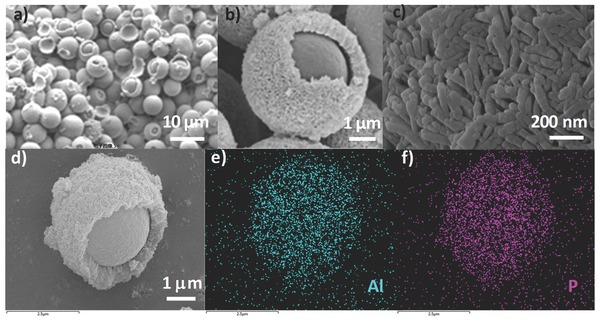
a) SEM image of ys‐AlPhPO; b,c) high‐resolution SEM images; d–f) energy‐dispersive X‐ray spectroscopy (EDX) mapping results of a single aluminum phenylphosphonate microsphere obtained by hydrothermal treatment for 3 h (ys‐AlPhPO).

**Figure 2:**
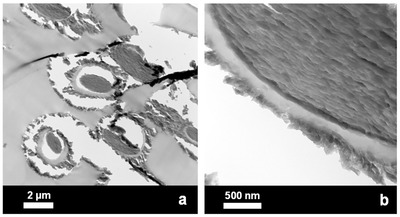
Transmission electron microscopy (TEM) images (ultramicrotomed sections) of ys‐AlPhPO microspheres: a) low magnification and b) high magnification.

The synthesis involves a facile template‐free hydrothermal process using urea as a precipitant, in the presence of phenylphosphinic acid (PPA, C_6_H_7_O_3_P) and aluminum nitrate to form yolk‐shell structured aluminum phenylphosphonate microspheres. Urea has been selected as a precipitating reagent because its hydrolysis is much slower, which in contrast to the precipitants such as NaOH and NH_3_·H_2_O, is well suited for precise control of the pH value needed for monitoring the morphology evolution. The morphology of the as‐synthesized YSNs material (ys‐AlPhPO) after 3 h of hydrothermal treatment (HT) was established by scanning electron microscopy (SEM; see **Figure** **1**). As can be seen in the panel (a) of this figure the sample is composed of uniform and well‐dispersed yolk‐shell structured microspheres with an average diameter of about 7 μm. The high‐resolution SEM (HRSEM) images (Figure 1b,c) further reveal that the surface of ys‐AlPhPO is relatively smooth, the 500 nm thick porous shells are disorderly self‐assembled by a large number of rod‐like nanoparticles with a size of 50 × 200 nm, and the size of hollow space between core and shell is around 200 nm. The energy dispersive X‐ray (EDX) mapping analysis (Figure 1d–f) indicates aluminum and phosphorus homogeneously distributed throughout the whole microsphere. The morphology of yolk‐shell structured microspheres is also confirmed by the transmission electron microscopy (TEM) image (**Figure** **2**).

The porous structure of ys‐AlPhPO microspheres is further evidenced by N_2_ sorption analysis (Figure S1, Supporting Information). The sample exhibits type IV adsorption–desorption isotherm with H3‐type hysteresis loop, which indicate the presence of mesopores, echoing with HRSEM results that the non‐uniform mesopores are formed by the disorderly assembled rod‐like nanoparticles. The Brunauer–Emmett–Teller (BET) specific surface area and total pore volume of the sample are calculated to be 163 m^2^ g^−1^ and 0.28 cm^3^ g^−1^, respectively. The powder X‐ray diffraction (XRD) pattern of ys‐AlPhPO (Figure S2, Supporting Information) exhibits a single strong diffraction peak at 2*θ* of 5.8°. The energy‐dispersive X‐ray spectroscopy (EDX) result of ys‐AlPhPO suggests that the Al/P ratio is 0.81. The inductively coupled plasma (ICP) and elemental analyses of ys‐AlPhPO indicate that the content of Al, P, C, and H is 9.20%, 12.78%, 33.03%, and 3.58%, respectively. The calculated Al/P ratio of 0.83 is in a good agreement with the result of the EDX analysis. As expected, the ^13^C magic angle spinning (MAS) nuclear magnetic resonance (NMR) spectrum shows peaks in the chemical shift range from 125 to 135 ppm representing resonance signals of the benzene ring (Figure S3, Supporting Information). The solid‐state NMR together with the FT‐IR data (Figure S4, Supporting Information) demonstrate the incorporation of phenylphosphonate into the framework through the coordination between aluminum and phenylphosphonate, and confirm that the phenylphosphonate can retain the structural integrity during the hydrothermal synthesis process.

### 2.2 Formation Mechanism

**Figure 3:**
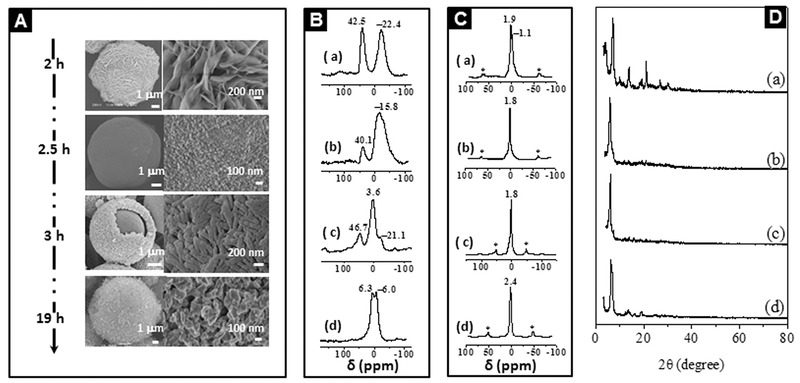
The SEM images, ^27^Al MAS NMR, ^31^P MAS NMR spectra, and powder XRD patterns of AlPhPO microspheres using hydrothermal reaction time from 2 to 19 h.

To investigate the formation mechanism of the hierarchical yolk‐shell structured aluminum phenylphosphonate microspheres through one‐step precipitation and hydrothermal synthesis process, a series of samples was synthesized by varying hydrothermal treatment time. The crystallization processes and morphological evolution as a function of HT time were monitored by SEM, ^27^Al MAS NMR, ^31^P MAS NMR, and powder XRD as illustrated in **Figure** **3**. When the HT was carried out for 2 h, the morphology of the resulting microspheres was flower‐like (Figure 3A). At higher magnification, one can clearly see that the nanosheets with sizes of 1–2 μm in length and 20–30 nm in thickness as petals are connected through a central core to form 3D flower‐like structure, and the assembly of petals is uniform and compact. The smooth‐surfaced microspheres without obvious branched structure can be observed for cs‐AlPhPO. A closer examination of these images shows that the surface of microspheres consists of spherical nanoparticles with diameter of 20–30 nm. When the HT is prolonged to 3 h, the morphology of the samples transforms to the yolk‐shell structured microspheres. Afterward, the resulting samples show spiny‐type microsphere morphology with porous hollow structure until 19 h of HT as evidenced by SEM and TEM imaging (Figure 3A and Figure S5, Supporting Information).

Apart from the morphological transformation of the samples, the variation of the composition and structure was also monitored as a function of HT time. Elemental analysis revealed that for HT time varying from 2, 2.5, 3 to 19 h, the Al/P ratio of the products increased from 0.67, 0.80, 0.83 to 2.33, respectively. The Al/P ratio of AlPhPO microspheres continued to increase with increasing HT time. The ^27^Al MAS NMR spectrum of cs‐AlPhPO synthesized for 2 h of HT exhibits two resonance peaks centered at 42.5 and −22.4 ppm (Figure 3B(a)), which can be assigned to tetra‐coordinated and hexa‐coordinated aluminum linked with phenylphosphonate, respectively. With the prolonged HT time, the ^27^Al MAS NMR spectrum of AlPhPO microspheres synthesized for 2.5 h of HT presents a less‐intense resonance peak centered at 40.1 ppm related to tetra‐coordinated aluminum and an intensive resonance peak centered at −15.8 ppm associated with hexa‐coordinated aluminum (Figure 3B(b)). This analysis indicates that the initially formed tetra‐coordinated aluminum centers further coordinate with hydroxyl groups in the reaction solution. When the HT time increases to 3 h, the ^27^Al MAS NMR spectrum of ys‐AlPhPO appears to have a new strong resonance peak centered at 3.6 ppm, in addition to two resonance peaks centered at 46.7 and −21.1 ppm assigned to tetra‐coordinated and hexa‐coordinated aluminum linked with phenylphosphonate (Figure 3B(c)), respectively, which may be due to hexa‐coordinated aluminum with unsymmetrical oxygen environment formed by the linkage of aluminum with hydroxyl besides phenylphosphonate.^7^, ^8^ When the HT time reaches 19 h, the ^27^Al MAS NMR spectrum of hs‐AlPhPO shows two new overlapped resonance peaks centered at 6.3 and −6.0 ppm, which can be attributed to octahedrally coordinated Al centers with different oxygen coordination environments. In short, the intensity of peaks related to tetra‐coordinated and hexa‐coordinated aluminum linked with phenylphosphonate decrease with prolongation of the HT time, and eventually these peaks disappear when the HT time reached 19 h. The intensity of the peak related to hexa‐coordinated aluminum with unsymmetrical oxygen environment connected with hydroxyls besides phenylphosphonates increases with prolonged HT time, and becomes stable octahedrally coordinated aluminum when the HT time approaches 19 h. The chemical shift variation in the ^27^Al MAS NMR spectra suggests the local environment of Al has been changed with prolongation of the HT time from 2 to 19 h, which is accompanied by the coordination of aluminum with hydroxyl groups. The ^31^P MAS NMR spectrum of AlPhPO microspheres indicates that phosphorus species in aluminum phenylphosphonate microspheres also change with prolonged HT time, suggesting that the coordination mode of phosphonate groups varies with the variation of local environment of aluminum (Figure 3C). The different diffraction patterns of AlPhPO microspheres synthesized using different HT times can be observed in Figure 3D, which shows that the crystalline phases of AlPhPO microspheres evolve with changing HT time from 2 to 19 h. The powder XRD data are consistent with the findings of elemental analysis and solid‐state NMR studies.

**Figure 4:**
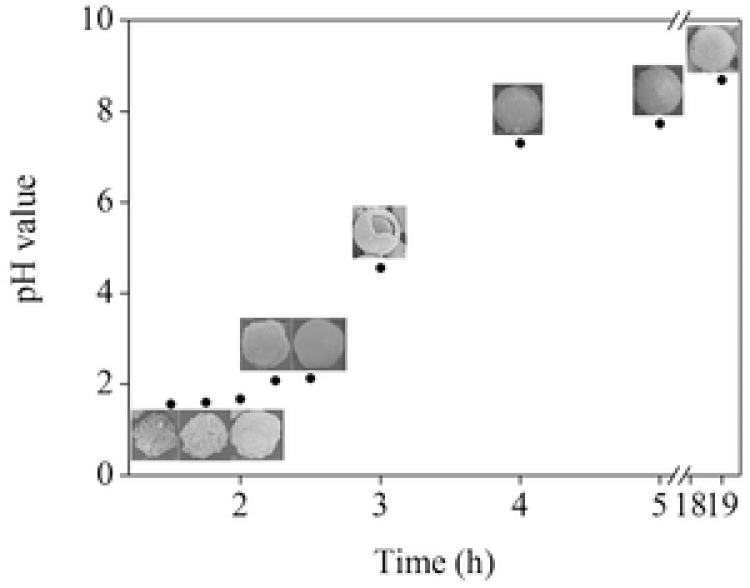
The morphological evolution of the aluminum phenylphosphonates for different pH values of the synthesis system tuned by hydrothermal treatment time.

Essentially, the transformation of morphology as well as the change of composition and structure with the prolongation of HT time result in the increase of pH value in the synthesis system. It is obvious to notice that the flower‐like microspheres and smooth‐faced microspheres without branched structure can only be obtained in a very narrow range of pH value from 1.6 to 2.1, while the spiny‐type microspheres with hollow structure can be synthesized in a pH range from 7.3 to 8.7. It is worthy to mention that the yolk‐shell structured microspheres are intermediates during the morphology transformation from smooth‐faced microspheres to spiny‐type microspheres with hollow structures accompanied by the sharp increase in the pH value from 2.1 to 7.3 (**Figure** **4**). Therefore, the morphology of aluminum phenylphosphonate can be precisely controlled by adjusting HT time, which directly determines the pH value of the synthesis system.

**Figure 5:**
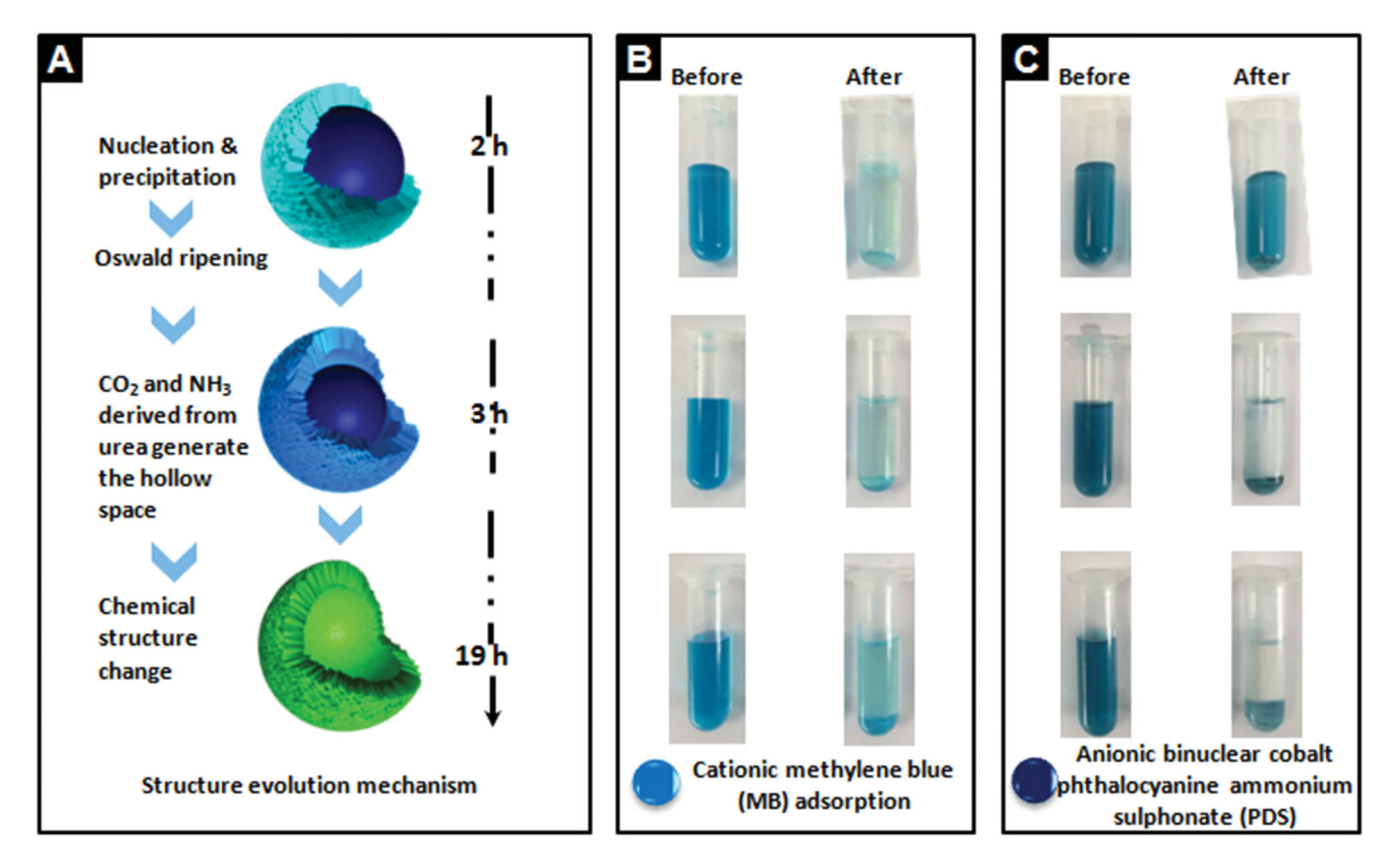
a) Schematic illustration of the morphological evolution of aluminum phenylphosphonate microspheres; b) cationic methylene blue (MB) and c) anionic binuclear cobalt phthalocyanine ammonium sulphonate (PDS) uptake profile photographs obtained by using different aluminum phenylphosphonate microspheres from core‐shell, yolk‐shell to hollow structures.

The plot of the zeta potential versus pH (Figure S6, Supporting Information) indicates that the electrostatic interactions between the cationic shell and the anionic yolk control the formation of these smooth‐faced microspheres. The formation mechanism is illustrated in **Figure** **5**; the flower‐like microspheres are formed at the beginning of the reaction within 2 h due to the nucleation and precipitation.^9^ The chemical structure of flower‐like microspheres is Al_2_(O_3_PC_6_H_5_)_3_⋅H_2_O, which is similar to that in the previous report.[[qv: 8d]] Further prolongation of the HT time to 2.5 causes an increase in the pH value from 1.6 to 2.1 in the synthesis system, which results in the conversion of the flower‐like microspheres to the smooth‐surfaced microspheres through an Oswald ripening. When the HT time reaches 3 h and the pH value increases to 4.6, the outside surface of the smooth‐surfaced microspheres is transformed to the mesoporous shell through an Oswald ripening and chemical structure variation, meanwhile the unconverted smooth‐surfaced microspheres act as a yolk to form yolk‐shell structures through electrostatic interactions. This can be evidenced by the fact that the smooth‐surfaced microspheres serve as the yolk with a negative potential of −4.1 mV and the spiny‐type microspheres resemble shells with a positive potential of +24.6 mV at pH 4.6. Increasing HT time to 4 h causes an increase in the pH value to 7.3 and the hydrolysis of aluminum forms Al(OH)_4_^−^ and Al(OH)_3_ in the reaction solution. At pH exceeding 5, the yolk is converted to the shell by consumption of Al(OH)_4_^−^ or Al(OH)_3_ leading to the formation of spiny‐type microspheres with hollow structures. Afterward, the shell of hollow‐structured microspheres grows inward and the cavity becomes thick with prolonged HT time. The morphological transformation of AlPhPO microspheres from flower‐like to smooth‐surfaced and yolk‐shell, and then to hollow structures is accompanied by the evolution of the chemical structure from Al_2_(O_3_PC_6_H_5_)_3_⋅H_2_O to Al_4_(O_3_PC_6_H_5_)_5_(OH)_2_⋅*n*H_2_O, and Al_5_(O_3_PC_6_H_5_)_6_(OH)_3_⋅*n*H_2_O, and finally to Al_7_(O_3_PC_6_H_5_)_3_(OH)_15_⋅*n*H_2_O as speculated on the basis of elemental analysis, solid NMR, and powder XRD data. Briefly, the morphological transformation of microspheres with prolonged HT time can be ascribed to: (1) structural change, more specifically, the change of aluminum coordination environment derived from tetra‐coordination aluminum further coordinated to hydroxyl with pH value increasing in the synthesis system, (2) Oswald ripening process, and (3) CO_2_ and NH_3_ derived from urea after prolonging the HT time serve as a bubble‐type template for formation of hollow space and porous structures. The morphological transformation from core‐shell to yolk‐shell and next to hollow spheres has also been observed in previous studies for different compositions.^10^

### 2.3 Adsorption of Dyes and Biomolecules

Very interestingly, these anionic cores and cationic shells of yolk‐shell structured aluminum phenylphosphonate microspheres (ys‐AlPhPO) can effectively uptake both cationic methylene blue (MB) and anionic binuclear cobalt phthalocyanine ammonium sulphonate (PDS) as shown in Figure 5B,C. The core‐shell structured microspheres (cs‐AlPhPO) with anionic dominant core can only uptake cationic MB, in contrast, hollow structured microspheres (hs‐AlPhPO) with cationic dominant shell are more effective for uptake of anionic PDS. In addition, adsorption of cationic MB and anionic PDS on ys‐AlPhPO microspheres at low pH value of 1.0 is better than that at high pH value of 9.0, indicating that acidic conditions are beneficial for adsorption of either cationic or anionic dyes (Figure S7, Supporting Information). The adsorption behavior of dyes indicates that these aluminum phenylphosphonate microspheres can be used for selective removal of dyes from mixtures, and further demonstrate the advantage of the Janus properties of yolk‐shell structured aluminum phenylphosphonate microspheres (ys‐AlPhPO) for adsorption‐based applications. The aluminum phenylphosphonate microspheres with amphoteric yolk‐shell structure can simultaneously adsorb anionic and cationic ions from solutions, suggesting that these materials would be excellent adsorbents for water treatment, drug delivery, peptide enrichment, etc.

**Figure 6:**
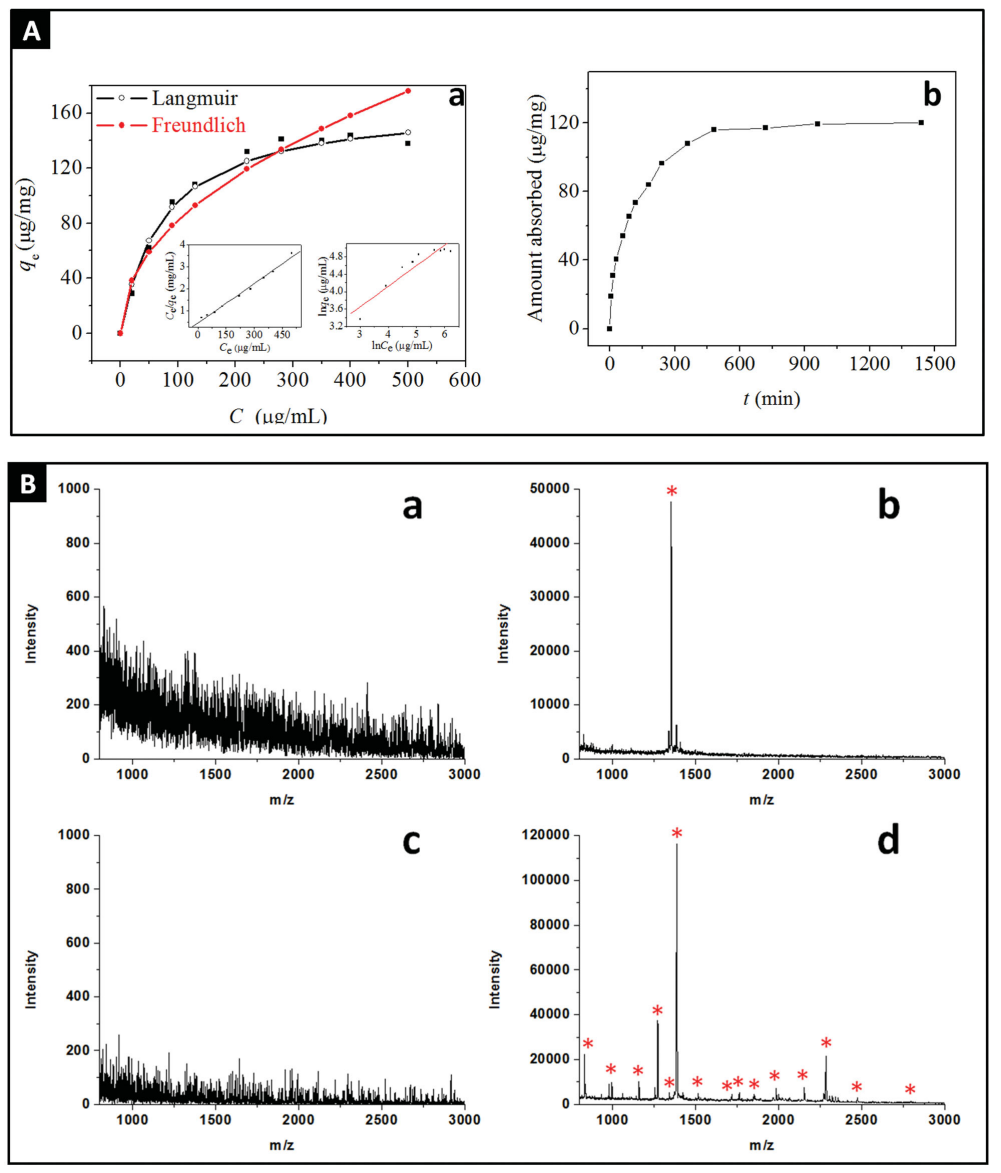
A) (a) Adsorption of salmon sperm DNA on ys‐AlPhPO (the scatter dots show experimental equilibrium adsorption data for salmon sperm DNA on ys‐AlPhPO microspheres; the black curves represent a fit of the experimental data by the Langmuir model; and the red curves represent a fit of the experimental data by using the Freundlich model); (b) adsorption kinetics of ys‐AlPhPO for salmon sperm DNA (200 μg of salmon sperm DNA mg^−1^ of microspheres; temperature = 25 °C). B) Enrichment tests based on the microspheres toward Alpha‐conotoxin IMI (a, b) and casein protein digests (c, d), where (a) and (c) are the control tests.

The promising application of these microspheres has been demonstrated for bio‐adsorption and enrichment; namely, ys‐AlPhPO microspheres exhibited an excellent performance for adsorption of salmon sperm DNA (**Figure** **6**A) and significant enrichment of low abundance phosphopeptides from casein protein digests (Figure 6B). Figure 6A(a) displays the equilibrium adsorption data of salmon sperm (ss‐DNA) on ys‐AlPhPO showing its high adsorption capacity of 140 μg mg^−1^. The equilibrium adsorption data were analyzed using two adsorption models, Langmuir and Freundlich models, in order to establish the most appropriate adsorption isotherm. The linear plots made for ss‐DNA on ys‐AlPhPO are shown in insets of Figure 6A(a). The left inset in Figure 6A(a) shows that the equilibrium adsorption data fulfills the linear form of Langmuir equation over the concentration range studied and the correlation coefficient is very high (Table S1, Supporting Information), suggesting that adsorption of ss‐DNA on ys‐AlPhPO can be accurately fitted by Langmuir equation derived for monolayer adsorption. There is a rather large deviation from linearity for ss‐DNA/ys‐AlPhPO adsorption system analyzed according to Freundlich equation, which is evidenced by poor correlation coefficient (see the right inset in Figure 6A(a) and Table S2, Supporting Information). The adsorption kinetics of ss‐DNA on ys‐AlPhPO was also studied as shown in Figure 6A(b). From the profiles of adsorption kinetics, ys‐AlPhPO microspheres can reach the adsorption equilibrium within 6 h. The adsorption kinetics experimental data obtained for salmon sperm DNA on ys‐AlPhPO were analyzed by applying two prevalently used kinetics models, pseudo‐first‐order and pseudo‐second‐order, the linear plots of which are displayed in Figure S8 (Supporting Information). The linear fitting of these data is very good as evidenced by the correlation coefficients close to unity (Table S3, Supporting Information), showing that both the pseudo‐first‐order and pseudo‐second‐order kinetics models can represent well adsorption of salmon sperm DNA on ys‐AlPhPO, although better correlation is observed for pseudo‐second‐order model. This result suggests that physical adsorption and chemical adsorption of ss‐DNA on ys‐AlPhPO may occur simultaneously.

The microspheres were also employed to enrich low concentration of biomolecules for matrix‐assisted laser desorption‐ionization time‐of‐flight mass spectrometry (MALDI TOF MS) detection. Alpha‐conotoxin IMI, a functional peptide (sequence GCCSDPRCAWRC) was used as model, which can selectively block alpha‐delta site of the muscle acetylcholine receptor. The signal strength of alpha‐conotoxin IMI can be raised to over 45 000 (Figure 6B(b)) after treatment with microspheres. The microspheres were further validated in the complex bio‐sample, where enrichment of phosphopeptides was performed toward casein protein digestion. As shown in Figure 6B(d), the signal of phosphopeptides cannot be identified at a concentration of 4.3 × 10^−9^ m from the MS spectrum; whereas the enriched phosphopeptides from the digests of the casein protein can be identified successfully after exposure to the microspheres. The above results indicate the strong potential of microspheres as promising alternatives for enrichment of low abundance peptides.

**Figure 7:**
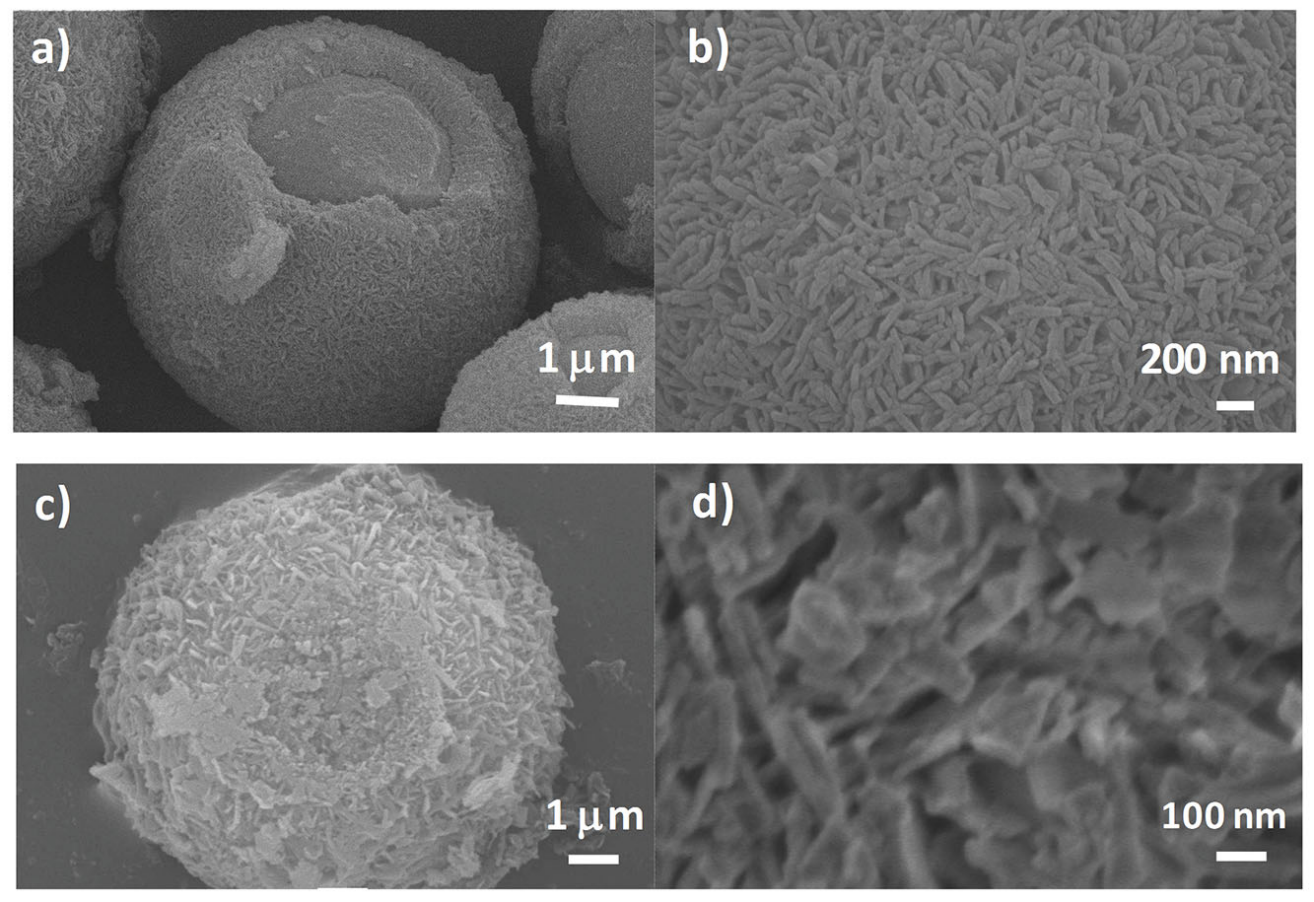
SEM images of inorganic microspheres (AlPO) obtained by calcination of a,b) ys‐AlPhPO and c,d) hs‐AlPhPO microspheres under air atmosphere at 550 °C for 2 h.

Furthermore, inorganic aluminum phosphate microspheres (AlPO) with the same morphology as their precursors, aluminum phenylphosphonate microspheres, can be obtained by calcination at 550 °C under air atmosphere for 2 h. No visible change in the particle size and structure was observed from SEM images (**Figure** **7**). At a high magnification, the calcined samples resemble disordered agglomerates of nanoparticles similar to those of their precursors.

## 3 Conclusions

In summary, a one‐step precipitation and hydrothermal method have been developed for the synthesis of amphoteric yolk‐shell structured aluminum phenylphosphonate microspheres with mesoporous shells. Aluminum phenylphosphonate microspheres with different surface morphologies, from flower‐like to smooth‐surfaced microspheres and yolk‐shell microspheres, and then to hollow structured microspheres, can be facilely obtained by varying the hydrothermal treatment time. The observed morphological transformation together with the change in the composition and structure of aluminum phenylphosphonate microspheres with reaction time can be attributed to the variation of Al coordination, evidenced by ^27^Al MAS NMR. The amphoteric yolk‐shell structured microspheres are formed by electrostatic interactions between the anionic yolk and cationic shell, and can be used for selective adsorption of dyes. The yolk‐shell structure and unique amphoteric properties make aluminum phenylphosphonate microspheres suitable for various applications in many fields such as environment, catalysis, biology, and nanomedicine.

## 4 Experimental Section

*Chemicals and Reagents*: All materials were of analytical grade and used as received without any further purification. PPA (C_6_H_7_O_3_P, 99%), aluminum nitrate nonahydrate, (Al(NO_3_)_3_⋅9H_2_O, ≥98%), α‐cyano‐4‐hydroxycinnamic acid (CHCA, ≥99%, matrix use only), trifluoroacetic acid (TFA, ≥99%), acetonitrile (ACN, ≥99%), and proteins/peptides were purchased from Sigma‐Aldrich. Salmon sperm DNA for adsorption and release was obtained from Wako Pure Chemical Industries Ltd. and used as received (the absorbance of 260 and 280 nm were measured and the *A*_260 nm_/*A*_280 nm_ ratio was about 1.8, indicating the high purity of salmon sperm DNA without contamination of protein). Other reagents were purchased from ShangHai Chemical Reagent Inc. of Chinese Medicine Group.

*Synthetic Procedure: x*‐AlPhPO microspheres were synthesized via a hydrothermal method using C_6_H_7_O_3_P and Al(NO_3_)_3_⋅9H_2_O in the presence of urea as the precipitation reagent. In a typical synthesis, C_6_H_7_O_3_P (0.3 mmol) and Al(NO_3_)_3_⋅9H_2_O (0.3 mmol) were dissolved into 15 mL of HNO_3_ solution (0.035 mol L^−1^) and then urea (15 mmol) was added into the above solution. The mixture was stirred for 30 min until a homogeneous solution was formed; next, the resultant solution was transferred into a Teflon‐lined autoclave and heated at 100 °C for *n* h (*n* = 1.5, 1.75, 2, 2.25, 2.5, 3, 5, and 19) under static conditions. After cooling to room temperature naturally, the solid products were collected using a membrane filter (0.22 μm) and washed with distilled water repeatedly, and dried at room temperature. The as‐synthesized materials were denoted as *x*‐AlPhPO, where *x* = cs, ys, and hs, refer to the core‐shell, yolk‐shell, and hollow structures, respectively.

*Characterization*: PXRD patterns were recorded on an Empyrean powder diffraction system using Cu Kα radiation of 0.15406 nm wavelength. The nitrogen adsorption experiments were performed at −196 °C on a Micromeritics ASAP 3000 system. Prior to the measurement, the samples were outgassed at 120 °C for at least 6 h. The BET specific surface areas were calculated using adsorption data in the relative pressure range of *P*/*P*_0_ = 0.05–0.25. Pore size distributions were calculated from the adsorption branch using the Barrett–Joyner–Halenda (BJH) method. The total pore volumes were estimated from the amounts adsorbed at a relative pressure (*P*/*P*_0_) of 0.99. HRSEM, field‐emission scanning electron microscopy (FESEM), and SEM images were taken on a HITACHI S‐5500, HITACHI SU 8010, and JSM 6360 LV microscope operating at an accelerating voltage of 1–30 kV, respectively. TEM imaging was performed using a JEOL JEM‐2010 at an acceleration voltage of 120 kV. FT‐IR spectra were collected with a TENSOR 27 IR spectrometer in the range of 4000–400 cm^−1^ using KBr pellet. ^13^C (100.5 MHz) cross‐polarization magic angle spinning (CP‐MAS), ^31^P (161.8 MHz), and ^27^Al (79.4 MHz) MAS solid‐state NMR experiments were recorded on a BRUKER DRX 400 spectrometer equipped with a magic angle spin probe in a 4 mm ZrO_2_ rotor. ^13^C signals were referenced to tetramethylsilane, ^31^P NMR signal was referenced to H_3_PO_4_ (85 wt%). The experimental parameters were 6 kHz spin rate, 2 s pulse delay, and 6 min contact time for ^13^C CP‐MAS NMR experiments; 8 kHz spin rate, 3 s pulse delay, and 10 min contact time for ^31^P MAS NMR experiments.

*Adsorption of Cationic MB and Anionic Binuclear Cobalt PDS*: cs‐AlPhPO, ys‐AlPhPO, and hs‐AlPhPO were dried at 80 °C for 24 h in a vacuum oven prior to adsorption of dyes. In each adsorption experiment, 100 mg of *x*‐AlPhPO were added to 3 mL of MB (0.01 mg mL^−1^) and PDS (0.05 mg mL^−1^) solutions, and the resulting mixture was continuously shaken in a shaking bath at room temperature for 12 h and then was standing for 12 h.

## Supporting information

As a service to our authors and readers, this journal provides supporting information supplied by the authors. Such materials are peer reviewed and may be re‐organized for online delivery, but are not copy‐edited or typeset. Technical support issues arising from supporting information (other than missing files) should be addressed to the authors.

Supplementary
