# Supplementary material for: Yolk–Shell‐Structured Aluminum Phenylphosphonate Microspheres with Anionic Core and Cationic Shell
Source: Adv Sci (Weinh). 2016 Feb 25;3(5):1500363. doi: 10.1002/advs.201500363 (PMC5069564; doi:10.1002/advs.201500363)
Supplement: Supplementary file 1 — Supplementary [file ADVS-3-0a-s001.pdf]

Copyright WILEY-VCH Verlag GmbH & Co. KGaA, 69469 Weinheim, Germany, 2016.

ADVANCED SCIENCE
Open Access

Supporting Information

for *Adv. Sci.*, DOI: 10.1002/advs.201500363

Yolk–Shell Structured Aluminum Phenylphosphonate Microspheres with Anionic Core and Cationic Shell

Liqiu Zhang, Kun Qian, Xupeng Wang, Fan Zhang, Xin Shi, Yijiao Jiang, Shaomin Liu, Mietek Jaroniec, and Jian Liu**

Supporting Information

© Wiley-VCH 2016

69451 Weinheim, Germany

Yolk-Shell Structured Aluminum Phenylphosphonate Microspheres with Anionic Core and Cationic Shell**

Liqiu Zhang, Kun Qian, Xupeng Wang, Fan Zhang, Xin Shi, Yijiao Jiang, Shaomin Liu, Mietek Jaroniec, and Jian Liu**

[*] L. Zhang, X. Wang, F. Zhang, Dr. X. Shi*
Institute of Chemistry for Functionalized Materials
School of Chemistry and Chemical Engineering, Liaoning Normal University
850 Huanghe Road, Dalian 116029, China
E-mail: xinshi@lnnu.edu.cn

Dr. K. Qian
School of Biomedical Engineering
Shanghai Jiao Tong University Med-X Research Institute
1954 Huashan Road, Xuhui District, Shanghai 200030, China

Dr. Y. Jiang, Prof. S. Liu, Dr. J. Liu*
Department of Chemical Engineering
Curtin University
Perth, Western Australia 6845, Australia
E-mail: jian.liu@curtin.edu.au

Prof. Dr. M. Jaroniec
Department of Chemistry and Biochemistry
Kent State University
Kent, Ohio 44242, United States

Experimental Section

Adsorption of salmon sperm DNA. *ys*-AlPhPO was dried at 80 °C for 24 h in a vacuum oven prior to adsorption of salmon sperm DNA. Adsorption isotherms were obtained by preparing a series of salmon sperm DNA solutions with concentration ranging from 2 to 440 $\mu g{\cdot}mL^{-1}$ in pure water. In each adsorption experiment, 5 mg of the *ys*-AlPhPO sample was added to 10 mL of salmon sperm DNA solutions with different concentrations, and the resulting mixture was continuously shaken in a shaking bath at room temperature for 24 h. The supernatant was separated from the solid material by repetitive centrifugation and the salmon sperm DNA content in the supernatant was measured using UV absorption at 260 nm.

The kinetics of salmon sperm DNA adsorption was investigated by suspending 5 mg of the sample in salmon sperm DNA solution (200 $\mu g{\cdot}mL^{-1}$, 10 mL). The mixture was shaken in a shaking water bath at room temperature for different intervals and measured periodically. For each measurement, the upper solution was separated from the solid material by repetitive centrifugation and the salmon sperm DNA content of upper limpid solution was measured using UV absorption at 260 nm.

The amount of salmon sperm DNA adsorbed on *ys*-AlPhPO was calculated as the difference in the concentration of salmon sperm DNA before and after adsorption according to the following equation: $M = [C_iV - C_eV]/m$, M ($\mu g{\cdot}mg^{-1}$) is the amount of salmon sperm DNA adsorbed per microgram of the material studied; C_i ($\mu g{\cdot}mL^{-1}$) is the initial concentration of salmon sperm DNA; C_e ($\mu g{\cdot}mL^{-1}$) is the equilibrium concentration of salmon sperm DNA in the upper solution after adsorption; V (mL) is the volume of salmon sperm DNA solution; m (mg) is the weight of the adsorbent material.

Adsorption models. Two adsorption models, Langmuir and Freundlich, were applied to select the most appropriate adsorption isotherm for the system studied. The Langmuir model is applicable for an ideal monolayer adsorption on a homogeneous surface. Namely, adsorption takes place on energetically homogeneous sites present on the adsorbent surface; all adsorption sites are identical and energetically equivalent; each site can accommodate only one molecule or atom; and the lateral interactions between adsorbed molecules are neglected. The adsorption capacity can be expressed by the following equation, where q_e is the solid-phase concentration of adsorbate at the equilibrium ($\mu g{\cdot}mg^{-1}$), C_e is the equilibrium adsorbate concentration in the aqueous phase ($\mu g{\cdot}mL^{-1}$), K_L ($mL{\cdot}mg^{-1}$) is Langmuir constant and q_m ($\mu g{\cdot}mg^{-1}$) is the monolayer adsorption capacity.

$$q_e = \frac{q_m K_L C_e}{1 + K_L C_e} \qquad (1)$$

A linear expression of the Langmuir equation can be represented by

$$\frac{C_e}{q_e} = \frac{1}{q_m K_L} + \frac{1}{q_m} C_e \qquad (2)$$

The Freundlich model can be applied for adsorption on heterogeneous surfaces and is expressed by the following equation:

$$q_e = K_F \times C_e^{\frac{1}{n}} \qquad (3)$$

where q_e is the solid-phase concentration of adsorbate at the equilibrium (μg·mg^{-1}), C_e is the equilibrium adsorbate concentration in the liquid phase (μg·mL^{-1}), K_F is the Freundlich parameter [mL$^{1/n}$ ·μg$^{1-1/n}$·mg^{-1}], and 1/n is the heterogeneity factor. A linear form of the Freundlich expression can be obtained by taking logarithms of eqn (3).

$$\ln q_e = \ln K_F + \frac{1}{n} \times \ln C_e \qquad (4)$$

Adsorption kinetics models. The most common kinetics models, pseudo-first-order and pseudo-second-order reaction rate equations, were used to investigate the adsorption kinetics experimental data. The pseudo-first-order equation is given as follows:

$$\ln(q_e - q_t) = \ln q_e - k_1 t \qquad (5)$$

where q_t is the amount adsorbed at a given time (μg/mg); q_e is the corresponding equilibrium adsorption (μg/mg); k_1 is the rate constant (1/min) and t is the time (min). The plot of ln(q_e – q_t) against t should give a straight line with slope – k_1 and intercept lnq_e.

The pseudo-second-order equation is expressed as follows:

$$\frac{t}{q_t} = \frac{1}{k_2 q_e^2} + \frac{t}{q_e} \qquad (6)$$

where q_t is the amount adsorbed at a given time (μg/mg); q_e is the is the corresponding equilibrium adsorption (μg/mg); k_2 is the rate constant (mg·μg^{-1}·min^{-1}) and t is the time (min). The k_2 and the equilibrium adsorption (q_e) can be obtained experimentally from the slope and intercept of the plot of t/q_t versus t.

I. Langmuir, *J. Am. Chem. Soc.* **1918**, *40*, 1362.
H. M. F. Freundilich, *Z. Phys. Chem.* **1906**, *57*, 385.

Enrichment Tests. Enrichment tests towards peptide and protein digests were performed using low concentrated solutions prepared by a step-wise dilution method. The microspheres were dispersed in water at a concentration of 10 mg·mL^{-1} and 10 μL of the slurry was directly added to the prepared solutions. Then the supernatant was removed by centrifugation after 10 min enrichment and the microspheres were collected. For matrix-assisted laser desorption/ionization time-of-flight mass spectrometry (MALDI TOF MS), the microspheres were incubated with 1 μL of matrix solution (10

mg/mL CHCA in TFA/ACN/water, 0.1%/49.9%/50%, v/v/v) and analyzed on the plain steel MALDI plate. The MS spectra were collected accumulating 500 laser shots at 10 different spots on Bruker Autoflex II Smartbeam system and no smooth spectra were used.

Table S1. Langmuir adsorption isotherm parameters for salmon sperm DNA on *ys*-AlPhPO.

	K_L (mL·mg^{-1})	R^2	q_m (μg·mg^{-1})
ys-AlPhPO	2.256	0.9945	167.3

Table S2. Freundlich adsorption isotherm parameters for salmon sperm DNA on *ys*-AlPhPO.

	K_F [mL$^{1/n}$ ·μg$^{1-1/n}$·mg^{-1}]	b_F	R^2
ys-AlPhPO	9.3010	0.4730	0.9399

Table S3. Adsorption kinetics constants for salmon sperm DNA on *ys*-AlPhPO.

	Pseudo-first-order model		Pseudo-second-order model	
	k_1 (1/min)	R^2	k_2 (mg·μg^{-1}·min^{-1})	R^2
ys-AlPhPO	5.44×10^{-3}	0.9907	1.22×10^{-4}	0.9991

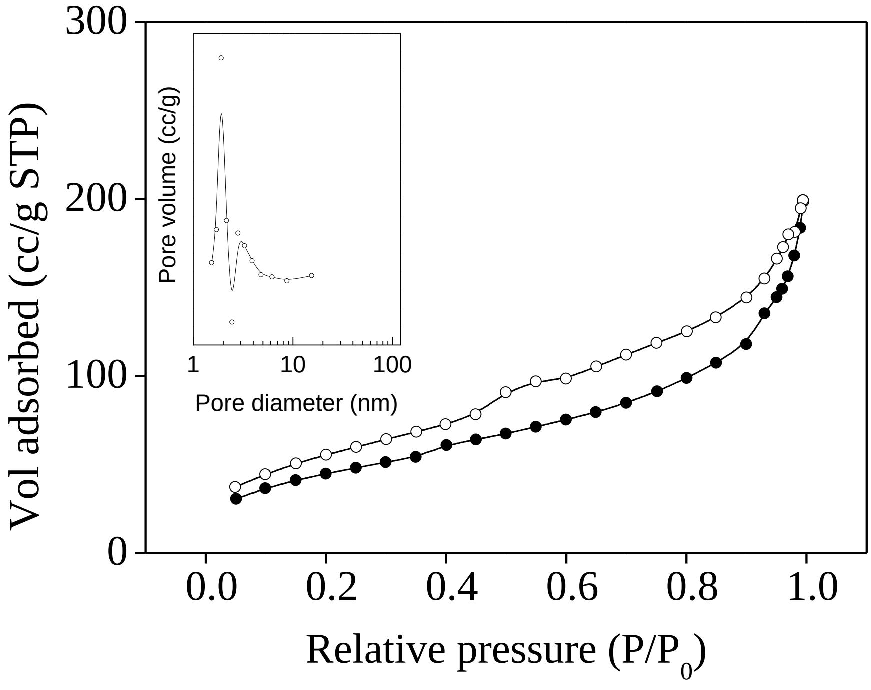

Figure S1. Nitrogen adsorption isotherm measured on *ys*-AlPhPO microspheres (inset shows the pore size distribution curve).

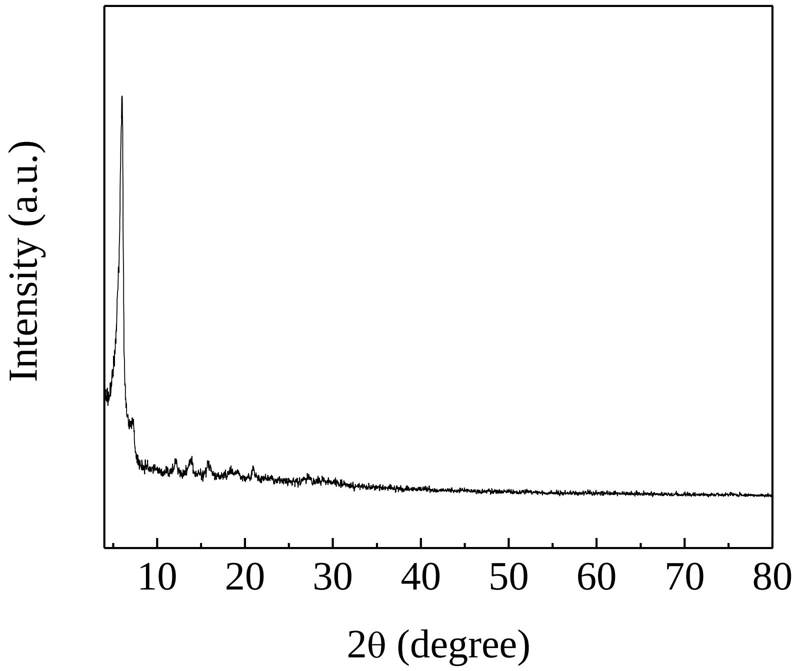

Figure S2. Powder XRD pattern of *ys*-AlPhPO microspheres.

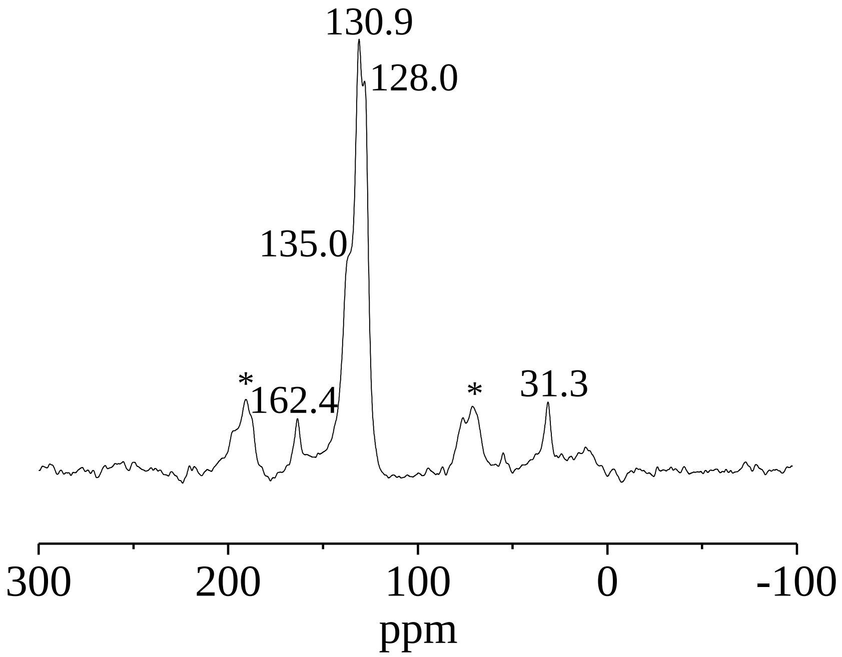

Figure S3. ^{13}C CP/MAS NMR spectrum of *ys*-AlPhPO microspheres. * Refers to the rotation side band.

The resonance signal at 128 ppm can be assigned to the quaternary carbon atom, in the *ipso* position, which is linked with phosphonate group. The resonance peaks at 130.9 ppm and 135.0 ppm can be attributed to carbon atoms in the *meta* and *para* positions, respectively. In comparison to the ^{13}C CP/MAS NMR spectrum of phenylphosphonic acid reported in the previous reference, in which there are four resonance signals located at 128.0, 129.7, 132.0, and 135.0 ppm corresponding to four different carbon atoms (*ipso*, *ortho*, *meta*, and *para* positions) in the benzene ring, Fig. S3 is consistent with the coordination of aluminum with phenylphosphonate in the sample resulting in the increase of steric hindrance of the *ortho* carbon atoms and the partial loss of conjugation between phosphonyl and phenyl. The resonance signal at 162.4 ppm is due to the carbon atom of carbonyl group in urea. Besides, the resonance peak observed at 31.3 ppm can be assigned to the carbon atom of methyl group in acetone, while the resonance signal at 207.3 ppm corresponding to the carbon atom of carbonyl group in acetone is overlaid by the rotation side band.

C. Gervais, M. Profeta, V. Lafond, C. Bonhomme, T. Azaïs, H. Mutin, C. J. Pickard, F. Mauri, *Magn. Reson. Chem.* **2004**, *42*, 445–452.

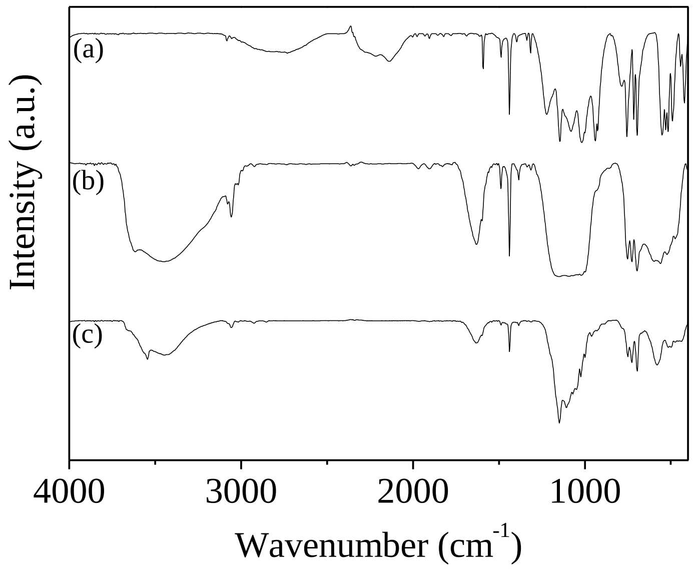

Figure S4. FT-IR spectra of (a) phenylphosphonic acid; (b) *ys*-AlPhPO microspheres; (c) *hs*-AlPhPO microspheres.

The strong and wide bands located in the range of 1000 cm^{-1}~1170 cm^{-1} are due to the Al–P–O stretching vibration, indicating that the materials studied were constructed by coordination of aluminum and phenylphosphonate species. The presence of characteristic bands at 1487 cm^{-1}, 1597 cm^{-1} and 3000 cm^{-1}~3100 cm^{-1} indicates that the samples contain benzene rings. The existence of sharp bands at 695, 725 and 750 cm^{-1} further illuminates that the benzene groups in the samples are monosubstituted. The strong and sharp band at 1438 cm^{-1} corresponding to the C–P stretching vibration is largely enhanced because of the direct connection of phosphorus atom with benzene ring. The strong band at 1630 cm^{-1} and broad band at 3445 cm^{-1} are associated with the O–H bending and stretching vibration of the adsorbed water, respectively. The disappearance of the band at 1222 cm^{-1} indicates the absence of a P=O bond in the framework of *ys*-AlPhPO and *hs*-AlPhPO microspheres.

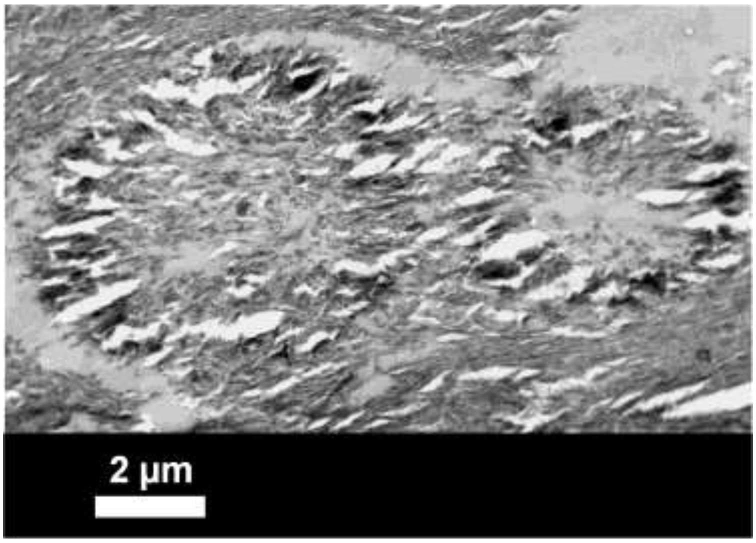

Figure S5. Transmission electron microscopy (TEM) images (ultramicrotomed sections) of *hs*-AlPhPO microspheres.

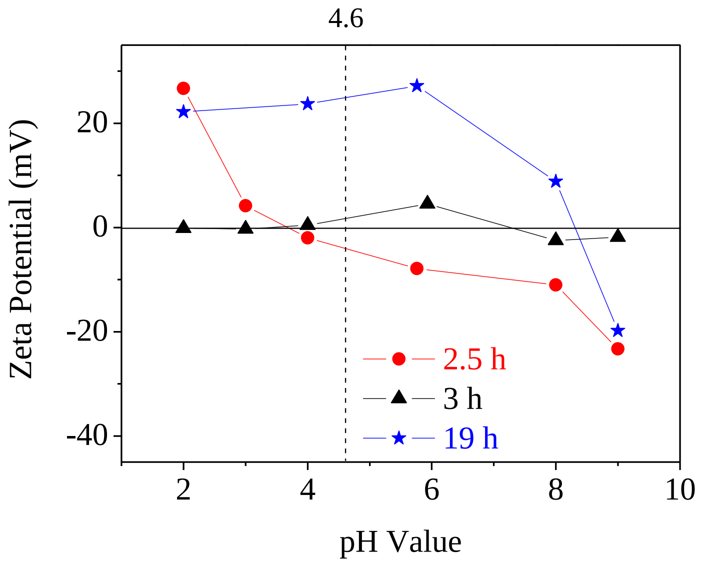

Figure S6. Zeta potential of *x*-AlPhPO microspheres at different pH values; the red, black and blue curves refer to the samples synthesized at HT conditions for 2.5 h, 3 h, and 19 h, respectively.

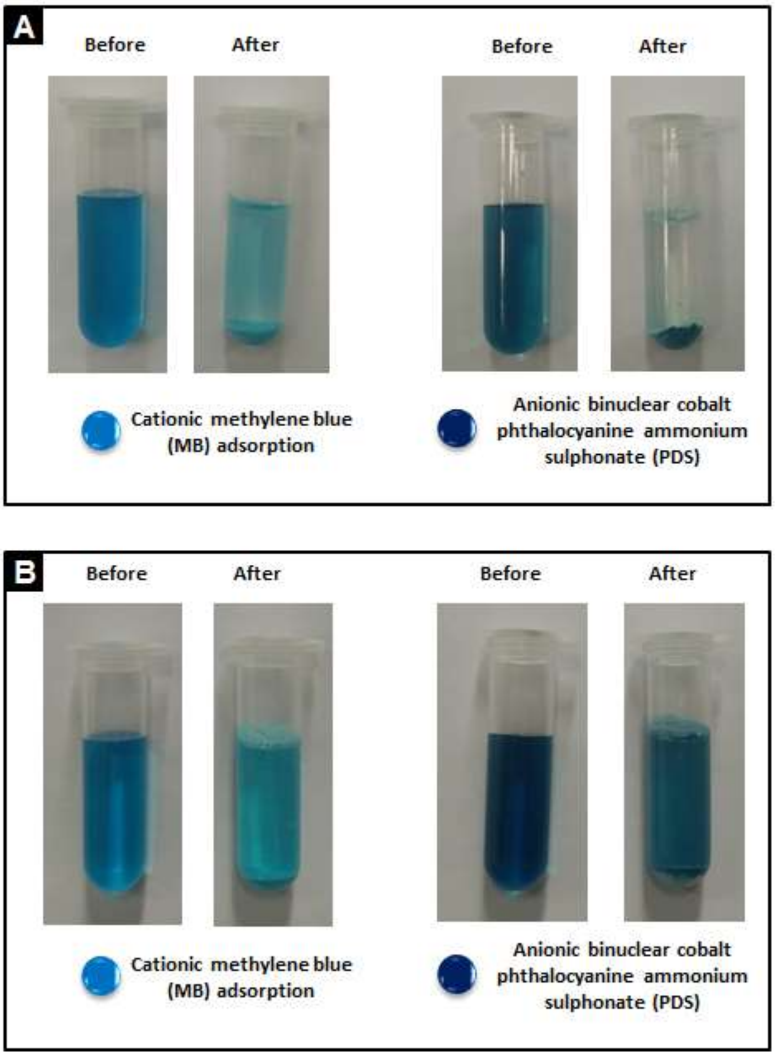

Figure S7. Adsorption behavior of *ys*-AlPhPO microspheres upon their exposure to cationic dye (MB) and anionic dye (PDS) at different pH values. A. pH = 1.0: (a) MS; (b) PDS; B. pH = 9.0: (a) MS; (b) PDS.

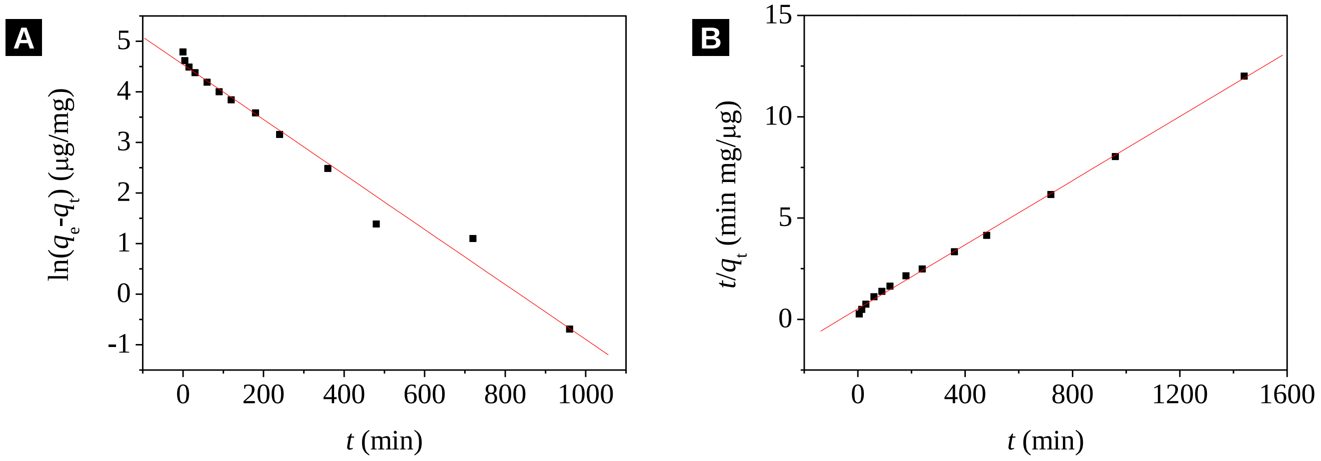

Figure S8. The linear plots representing data for salmon sperm DNA on *ys*-AlPhPO obtained by using (a) pseudo-first-order and (b) pseudo-second-order adsorption kinetics models.
